# Intemperate Physicians

**Published:** 1844-06

**Authors:** 


*Intemperate Physicians.*—Happily for society, the old race of tippling physicians has gone out of fashion. Formerly it was one of the common reproaches brought against the profession, that many of the best practitioners were intemperate. If they could be caught while sober, their advice was of the highest value. There is no occasion, in this age of soberness, for watching an opportunity to get medical advice from that class of physicians. In New England, there are a few drinking ones left—laudanum-taking men, perhaps, who vainly imagine that they are securely deceiving the world in regard to their habits, when the fact is, no one is in the dark but themselves.

How disgraceful—and, above all, what a fearful responsibility—to prescribe for the sick when muddled with liquor or stupefied with opium! The public have a vigilant eye on the remnant few of these semi-sober physicians, who must either conform to the requirements of reason and the temperance reformation, or go down in poverty and neglect to that low obscurity to which a moral community will certainly consign them.—*Ibid.*

